# Impact of a Medication Adherence Packaging Service on Patient-Centered Outcomes at an Independent Community Pharmacy

**DOI:** 10.3390/pharmacy9010011

**Published:** 2021-01-05

**Authors:** Catherine Phi, Lucas A. Berenbrok, Joni C. Carroll, Ashley Firm, Melissa Somma McGivney, Kim C. Coley

**Affiliations:** 1 Asti’s South Hills Pharmacy, Pittsburgh, PA 15234, USA; catbphi@gmail.com (C.P.); alf158@pitt.edu (A.F.); 2 School of Pharmacy, University of Pittsburgh, Pittsburgh, PA 15261, USA; berenbro@pitt.edu (L.A.B.); joni.carroll@pitt.edu (J.C.C.); mmcgivney@pitt.edu (M.S.M.)

**Keywords:** medication adherence, quality of life, community pharmacy, pharmacy services

## Abstract

The purpose of this project was to evaluate the impact of a comprehensive medication adherence packaging (RxMAP) service on patient medication-taking behaviors and patient-centered outcomes. Adult patients who utilized a single independent community pharmacy, enrolled in the RxMAP service for at least two consecutive cycles, and managed their own medications were eligible. The RxMAP service consists of multi-dose blister packaging in 28-day cycles, medication synchronization, monthly touchpoint calls, and delivery/mailing. A 13-item telephonic survey was administered, and patients’ verbal responses were captured by audio-recording and detailed note taking. Descriptive statistics were used to quantify the results and illustrative quotes representing the interview domains were selected. There were 42 patients who completed the survey: 88% reported they missed fewer doses compared to before using RxMAP; 71% were more likely to take their medications on time each day; 86% were more confident with managing their medications; and 74% were more independent. Finally, 64% of patients stated their overall quality of life was better now compared to before using the packaging service. These results demonstrate that medication adherence packaging services can positively impact patients’ medication-taking behaviors, increase their confidence in medication management, and improve perceived quality of life.

## 1. Introduction

Medication adherence plays an important role in the prevention and effective management of many chronic medical conditions. Medication nonadherence continues to be a significant problem in healthcare, contributing to morbidity, mortality, and avoidable healthcare costs [1,2,3]. Factors affecting medication adherence are complex but can be broadly categorized as either intentional or unintentional. Intentional non-adherence occurs when an individual makes a conscious decision, often due to motivation or their beliefs, not to take their medication as prescribed [4]. Medication cost, actual or perceived side effects, and patient beliefs about medication effectiveness are all factors that can lead to intentional nonadherence. Nonadherence related to a person’s ability to take their medication (e.g., difficulty opening a prescription bottle), confusion over their medication regimen, or forgetfulness can be classified as unintentional nonadherence [4].

Medication adherence is typically measured and assessed using surrogate markers of medication-taking, such as medication-possession-ratio (MPR) or proportion-of-days covered (PDC) [5]. PDC is a commonly used metric to assess performance as part of value-based models. Both MPR and PDC calculate adherence using the number of days a patient is thought to have medication on hand, based on quantity dispensed to the patient, over a period of time. However, there are significant limitations to using PDC and MPR markers, as each is only able to measure how much drug is provided to the patient. These measures provide limited insights into the patient’s true medication-taking behavior or experience, and fail to measure whether the medication was consumed by the patient.

Pharmacists play the leading role in supporting medication adherence. Several strategies community pharmacists utilize to improve adherence include medication synchronization, screening and brief interventions, and reminder calls [6,7,8,9]. Medication adherence packaging is another rapidly growing enhanced service offered by community pharmacies nationwide. These services often include packaging medications as either single or multi-dose packs, along with synchronization, which allows for all medications to be filled at the same time each month [10]. These medication packs are organized to aid the patient with the appropriate days and timing, such as morning, noon, dinner, and bedtime dosing, to simplify medication administration. Many community pharmacies that provide medication adherence packaging services also include regularly scheduled contact points (e.g., telephone calls) with patients to identify and resolve medication-related problems and confirm the patient’s current medication regimen. Adherence packaging has been shown to improve medication adherence as measured by MPR or PDC [11,12,13].

Medication adherence packaging programs have the potential to influence important patient-centered outcomes, such as increased independence, by relieving patients of the burden of managing multiple medications, a common source of intentional and unintentional nonadherence. However, to date there are limited published assessments of how medication packaging services can influence patients’ medication-taking behaviors and patient-centered outcomes [14]. The objective was to evaluate the impact of a comprehensive medication adherence packaging service on patient medication-taking behaviors and patient-centered outcomes.

## 2. Materials and Methods

Patients 18 years of age and older who were enrolled in the prescription medication adherence packaging (RxMAP) service at Asti’s South Hills Pharmacy at the time of the project initiation were eligible for participation. Asti’s South Hills Pharmacy is a high volume, single location independent pharmacy in Pittsburgh, Pennsylvania. This pharmacy offers a variety of enhanced patient care services including a medication synchronization program, comprehensive medication reviews, and point-of-care testing. The RxMAP service consists of multi-dose blister packaging in 28-day cycles, medication synchronization, monthly touchpoint telephone calls with patients, coordination of care between healthcare providers and case managers, and delivery/mailing. At the time of this project, approximately 400 patients were enrolled in RxMAP. Additional inclusion criteria were that (1) patients had to receive at least two consecutive cycle fills of medication through RxMAP, and (2) patients managed/coordinated their own medications at the time of the project.

This project utilized a telephonic survey administered from February through April 2019 by the primary investigator (C.P.). RxMAP patients were invited to participate in the survey during their regularly scheduled monthly touchpoint call. These monthly calls are typically used to inquire about changes in each patient’s medication regimen. The primary investigator confirmed patient eligibility, using the inclusion criteria noted previously, before administering the survey. Patients were then asked to think about their medication-taking experience while enrolled in RxMAP and compare that experience to before participating in RxMAP. The survey consisted of 13 questions and included six Likert questions and seven open-ended questions (Appendix A). Questions were informed by the Patients’ Lived Experience with Medicines (PLEM) framework using three broad domains of medication-related burden, medication-related beliefs, and medication-taking practice [15]. The PLEM conceptual model can be used to guide patient-centered assessments of medication adherence services and their impact on patient outcomes [15]. A fourth domain was added to provide information on the impact of the RxMAP program on patients’ perceived quality of life. Questions were developed by the primary investigator (C.P.) and reviewed for clarity and content by the study team.

Patients’ verbal responses to the survey questions were captured by audio-recording and detailed note taking by the primary investigator. Additionally, verbal responses to the Likert questions were captured by the primary investigator using Qualtrics™ (Provo, UT, USA) survey system. Audio-recordings of the open-ended questions were transcribed for fidelity of meaning. Descriptive statistics were used to quantify the results of the Likert questions. The project team met over several weeks to review the transcriptions and investigator-generated notes from the open-ended questions. The team identified illustrative quotes most representative of each PLEM domain and quality of life. This project was classified as program evaluation by the University’s Institutional Review Board.

## 3. Results

There were 250 patients that met inclusion criteria for the study. Of those eligible, 175 patients were contacted at least once by the primary investigator to participate in the survey. A total of 42 patients (24% response rate) completed the survey by the end of the study period. Their mean age was 64 (range 37–90) years with females representing 66% of participants. Patients received an average of 11 medications and were enrolled in RxMAP for a mean 27 months. Patients received their adherence packaged medications through the following mechanisms: 24.4% by mail; 56.1% by delivery driver; and 19.5% by in-person pharmacy pick up.

Patients were asked about the impact of the RxMAP program on their ability to remember medications and ability to take medications on time. Eighty-eight percent (n = 37) of patients reported they missed fewer doses compared to before using RxMAP, and 71% (n = 30) reported they were more likely to take their medications on time each day. Patients were asked to explain the impact of adherence packaging on their ability to take their medications as prescribed. The following are quotes that illustrate the impact of RxMAP:

*“I take [my medications] on a regular routine basis now; before it was haphazard. I knew I wasn’t taking them like I should.…It is more convenient to take them now; whereas [before] I would be missing [doses].”*

(Patient #11)

*“It’s so much easier; I know I’m not going to miss a pill. I don’t have to sit and fill all my bottles. You know, it’s much more convenient.”*

(Patient #17)

*“I take them on time now. Before, I would take them sporadically throughout the day instead of taking them all at once.”*

(Patient #12)

When patients were asked about a change in confidence in managing their medications, 86% (n = 36) stated they were more confident after RxMAP enrollment. Seventy-four percent (n = 31) of patients reported they were more independent after using medication adherence packaging.

*“It has brought my confidence in taking my medications up to 100%. I don’t need anybody to check behind me.”*

(Patient #38)

*”...it makes me feel more organized and more confident; I can look back and I can ask myself, did I take this?”*

(Patient #19)

*“I don’t need a person to come and do this for me anymore and [my fiancé] doesn’t need to take care of me.”*

(Patient #7)

Sixty-four percent (n = 27) of patients surveyed reported their overall quality of life was greater compared to before using the packaging service, with the remaining 36% (n = 15) stating that quality of life remained the same. The following quotes are representative of patients’ remarks on how their quality of life has been impacted by the service:

*“I was in and out of the hospital constantly; my lungs were filled with fluid and now I’m able to stay on [my medications] and take them on time each night.”*

(Patient #4)

*“Taking my medication how it is prescribed has helped me keep away infections and not be sick. Whenever I wasn’t able to [be] set up [with RxMAP] and was relying on other people and would forget, I would get sick and find out later that something was missing.”*

(Patient #10)

*“[My quality of life] is better because if I skip [my doses] I could go down the tube. I don’t want to be in a depressed state—I had too many.”*

(Patient #30)

Lastly, participants were asked about their general engagement in their healthcare now compared to before use of the RxMAP service. Fifty-seven percent (n = 24) of patients reported that they were more engaged in their healthcare since enrolling in the RxMAP service. There were 17 patients (40%) and 1 patient (2%) that reported no change or being less involved in their healthcare, respectively. Patients described their healthcare now in the following ways:

*“I feel more involved because [the RxMAP service] gets me more involved. I never looked at this stuff before. Now, I pay attention to what I am taking.”*

(Patient #7)

*“Right now, I am more concerned [with my medications]: my doctors ask me what I’m taking and I have it written down. I’m more concerned with what I am taking, morning and night.”*

(Patient #18)

## 4. Discussion

This project evaluated patient-reported experiences with a medication adherence packaging service at an independent community pharmacy. Specifically, this work assessed the impact of the RxMAP program on patients’ self-reported medication-taking behaviors, confidence in managing their medications, and perceived quality of life. The results provide new evidence that a patient’s ability to remember to take medication regimens, confidence in managing medications, independence, and perceived quality of life were all positively impacted by the adherence packaging service. Prior studies looking at medication adherence packaging have focused largely on the ability of packaging to impact surrogate adherence measures, specifically increasing PDC and MPR [11,13]. Although these measures provide important information about medication on hand, they provide little insight on patients’ actual medication-taking behaviors. Our study, however, found that 88% of RxMAP patients reported missing fewer doses and 76% reported they were more likely to take their medications on time. These self-reported improvements in medication-taking can have a significant impact on patient outcomes.

Previous studies demonstrate that increasing medication adherence positively impacts clinical outcomes such as blood pressure and asthma control [2,11,16,17,18]. This project adds to what is known about medication adherence by demonstrating that the use of medication packaging services can also improve patient-centered outcomes such as the ability to manage medications independently. Seventy-six percent of our participants reported they were more independent after enrolling in the medication adherence packaging service. Patients who have difficulty organizing and self-administering their medications often rely on family members or other caregivers to help with medication management, which can cause significant mental and financial burden to the caregiver [19,20,21]. In addition to greater independence, patients enrolled in the RxMAP program also reported greater confidence in managing their medications which may ultimately lead to better control and management of costly chronic disease states [16,17].

To offer medication adherence packaging services, pharmacies typically incur costs to make pharmacy staff available for patient telephone calls, prescription verification, and packaging. These costs may be reimbursed by value-based or performance-based models once the positive outcomes of adherence services are demonstrated to payers, though this model is not yet widespread [22]. Patients’ satisfaction with their healthcare services is also increasingly relevant to payers. The qualitative data from this research describing impact on improved adherence play a critical role in that conversation. As the United States’ healthcare environment moves toward value-based care, it is essential for pharmacists to demonstrate how the additional costs of their services can be offset by having a positive impact on important patient-centered outcomes. This information from this project can be used by pharmacies and pharmacy networks along with existing PDC-focused adherence data to leverage expansion of medication adherence packaging services through value-based payment opportunities.

There were some limitations to this work. Since patients were asked to reflect on their medication experiences and how they have changed since using RxMAP, there is the potential for recall bias. Patients may not have precisely remembered their prior medication-taking experiences. Additionally, the patient interviews were conducted by an employee at the pharmacy, which could have influenced the patients’ responses. Another limitation is the sample size and use of a single pharmacy location for the RxMAP service. Enrolling more patients and patients from multiple pharmacies would have increased the diversity of the patient population and strengthened the findings.

## 5. Conclusions

Medication adherence packaging services can positively impact patients’ medication taking behaviors, increase their confidence in medication management, and improve their perceived quality of life. Community pharmacies that provide medication adherence packaging services may contribute to improved patient health outcomes.

## Data Availability

The data presented in this project are available on request from the corresponding author. The data are not publicly available at the request of the partnering pharmacy.

## Appendix A. Survey Questions

1. Compared to before using the medication packaging service, how often do you miss doses of medication now? a. I miss fewer doses of medication than I did before. b. I miss about the same amount of doses as I did before. c. I miss more doses of medication than I did before. 2. Can you tell me about how the medication packing service has impacted how you remember to take your medications? _____ 3. Compared to before using the medication packaging service, how would you rate your ability to take your medications at the same time each day? a. I am less likely to take medications on time than I did before. b. I am equally as likely. c. I am more likely to take medications on time than I did before. 4. Can you tell me about how the medication packing service has impacted your ability to take your medications on time? _____ 5. Compared to before using the medication packaging service, how would you rate your overall confidence in being able to manage all of your medications now? a. I am less confident than I was before. b. I have the same amount of confidence as I did before. c. I am more confident than I was before. 6. Can you explain in what ways your confidence in managing your medications has been impacted by using the medication packs? _____ 7. Compared to before using the medication packaging service, how independent do you currently feel in managing your own medications? a. I am less independent than before. b. I have the same amount of independence. c. I am more independent than before. 8. In what ways has using the medication packaging service impacted your independence? _____ 9. Compared to before using the medication packaging service, how involved do you feel with your health care now? a. I feel less involved than I did before. b. I feel the same amount of involvement that I did before. c. I feel more involved than I did before. 10. Tell me more about how you feel your healthcare is now. _____ 11. Compared to before using the medication packaging service, how would you rate your overall quality of life now? a. My quality of life is poorer than before. b. My quality of life is the same than before. c. My quality of life is better than before. 12. How do you believe the medication packaging service has affected your quality of life? _____ 13. In what ways have your relationship with your pharmacy and pharmacy team changed? _____
